# Level of knowledge, attitude, and practice of family planning and associated factors among disabled persons, north-shewa zone, Amhara regional state, Ethiopia

**DOI:** 10.1186/s40834-020-00111-y

**Published:** 2020-07-01

**Authors:** Alemayehu Gonie Mekonnen, Alebachew Demelash Bayleyegn, Yared Asmare Aynalem, Tigist Demssew Adane, Mikyas Arega Muluneh, Meaza Asefa

**Affiliations:** 1 Department of Nursing, College of Health Science, Debre Berhan University, Po. Box 445, Debre Berhan, Ethiopia; 2 Department of Midwifery, College of Health Science, Debre Berhan University, Po. Box 445, Debre Berhan, Ethiopia; 3 Department of Medical Laboratory, College of Medicine, Debre Berhan University, Po. Box 445, Debre Berhan, Ethiopia

**Keywords:** Family Planning, Contraceptive, Disability, Debre Berhan

## Abstract

**Background:**

In Ethiopia, people with disabilities face socioeconomic disadvantages and they have a limited access to sexual and reproductive health information including family planning service. At present, however, there is a scarcity of research on the association between disability and family planning, and only limited data is available for disabled people in Ethiopia. Hence, this study assessed the level of knowledge, attitude, and practice of family planning and associated factors among disabled persons in North-shewa zone, Amhara regional state, Ethiopia.

**Methods:**

A cross-sectional survey was conducted from June to October 2019. A total of 397 study participants were interviewed using a structured and pre-tested questionnaire. A multistage systematic sampling technique was employed to select study participants. Data were entered into Epi data and exported into Statistical Package for the Social Sciences (SPSS) version 21 for analysis. Logistic regression was performed to analyze the data. A significant association was declared at a *p*-value of less than 0.05.

**Results:**

Forty-six percent of study participants were knowledgeable about family planning methods. The injectable was the most known method of modern contraception (74.8%) while withdrawal (18.1%) was the least known traditional family planning method. Fifty-five percent of our study participants had a good attitude about family planning methods and one-fourth (24.5%) of disabled persons currently utilized any method of family planning. Those having a good knowledge of family planning were 1.6 times more likely to utilize family planning methods than those having poor knowledge of family planning methods (AOR = 1.61, CI = 1.27, 16.24). Moreover, participants who completed college education were 7 times more likely to have a good knowledge of family planning methods than uneducated participants (AOR = 7.23; 95% CI = 2.28, 22.06).

**Conclusions:**

In this study, the knowledge, attitude, and practice of disabled people about family planning methods were relatively low. Due attention should be given to ensure that disabled people are well informed about family planning methods through information, education, and communication activities.

## Introduction

Family planning (FP) is an effective way of controlling fertility within a human rights framework by giving couples the ability to have their desired family size [1, 2]. Family planning has a significant role in improving the health of the mother and the child by avoiding undesired pregnancies and abortions, thus reducing the maternal and child mortality rate [3, 4]. However, social inequality, religious/cultural barriers, weak coordination across sectors, inadequate quality assurance actions, and misconceptions about modern contraceptives were reported as the challenges for family planning implementation [1, 5–7].

Specifically, persons with disabilities are marginalized groups of the populations and they frequently marginalized from FP education due to misconceptions that they are not sexually active [8–10]. Furthermore, disabled people may be subjected to unsafe abortion due to long-standing stigmatization [11]. They have poorer health outcomes than non-disabled people as a result of less access to reproductive health information. Healthcare services also lack sign language interpreters and other information formats such as Braille, audio, or plain language which can provide advice on sexual health-related issues including FP methods [12]. Remarkably, they were often unable to access community meetings about FP services [13]. Moreover, their disabilities may limit their chances to interact with non-disabled peers that can be the best opportunity for informal learning about sexual health and family planning [14].

Although disabled people have the same reproductive health needs as the abled people and they want to space and limit the number of children to their economic capacity [15], they are notably absent from equitable reproductive health access [16] and face barriers to information [17]. Health facilities lack physical infrastructure, suitable and affordable transportation, and assistive devices for persons with disabilities [11, 18]. As such, disabled people face unique barriers to accessing family planning services and are often treated as a low priority for those services. They are often socially isolated and abused which creates obstacles to obtaining FP services [12]. Stigma and negative attitudes by healthcare providers towards disabilities have been commonly cited as the barriers to access and uptake of family planning for disabled people [11, 19]. The previous study also reported a variety of factors that affect access to and uptake of family planning among people with disabilities. For example, research from four African countries found a lack of knowledge about FP methods and fear of side-effects are issues affecting access to and uptake of family planning among people with disabilities [20].

In Ethiopia, people with disabilities face socioeconomic disadvantages and they have a limited access to sexual and reproductive health information including family planning services. They have an increased risk of SRH-related problems and have become the main focus in recent years. At present, however, there is a scarcity of research on the association between disability and family planning, and only limited data is available for disabled people in Ethiopia. Hence, this study assessed the level of knowledge, attitude, and practice of family planning and associated factors among disabled persons in North-shewa zone, Amhara regional state, Ethiopia. An examination of the knowledge and attitude towards FP and the factors which influence their attitude will guide the effective usage of FP services for disabled people who are marginalized groups of the populations.

## Methods and materials

### Study design, setting and period

A cross-sectional survey was conducted from June to October 2019 in the North-shewa zone, Amhara regional state, Ethiopia. The Debre Berhan town (the zone city) is located at 130-k meter far from Addis Ababa (the capital city of Ethiopia). An estimated 214,595 people with any form of disability are estimated to live in the North-shewa zone. The zone has 10 functional disability-support organizations: one is found in Debre Berhan town and nine are found in the other nine districts of the zone. The rest 15 districts have no functional disability support-organization. Overall, these disability-support organizations comprised a total number of 1500 disabled persons. These organizations support all disabled peoples (physically handicapped, hearing impairment, partial mental impairment, visual impairment, multiple impairments). The support organizations provide advocacy service, provide life skill training and support to live in the community, get involved in work (*unpublished zonal health department report, 2019).*

### Study population

All the reproductive-age groups of disabled people who enrolled in the disability-support organizations in the north-shewa zone were the study participants. Those who were ill and unable to communicate due to being ill were excluded from the study. Dual disabilities such as unable to see plus unable to hear were also excluded from the study.

### Sample size and sampling technique

The sample size was calculated using a single population proportion formula. The assumptions were: the proportion of disabled persons who had reproductive health knowledge as 79.6% (*p* = 0.796) taken from Tanzania [16], with 95% confidence interval (CI) to be 1.96, and margin of error to be 5%. Adding a non-response rate of 5% and the design effect of 1.5, a total sample size of 412 persons with disabilities were selected.

A multistage systematic random sampling technique was employed. In the first sampling unit, five disability-support organizations were selected randomly. In the second sampling unit, the lists of disabled people (registry) were used as the sampling frame. The registry contained the contact addresses of disabled people. The calculated sample size was proportionally allocated to disability-support organizations and the required numbers of participants were selected using a systematic random sampling technique.

### Data collection

A pre-tested and structured questionnaire was used to collect the data. The questionnaire was designed in English then translated into Amharic (native language) and back into English to ensure consistency. The data were collected by trained enumerators through a face-to-face interview. Five data collectors and five supervisors participated in the study. One of the data collector was a certified sign language interpreter who could collect data from participants who have a hearing impairment. Data completeness were checked by the investigators and supervisors.

### Measurements

Most of the questions were taken from the Ethiopian demographic health survey 2016 [21] and peer-reviewed literature [14, 22]. The questions comprise the following sections; socio-demographic characteristics and questions which examined the participant’s knowledge, attitude, and practice of FP methods. Knowledge questions were: what is family planning? Which FP methods did you know? What are the side effects of using family planning? Respondents answered either *“Yes”* or “*No”* or *“Do not know”* from the listed options. The attitude of respondents towards FP methods was assessed with seven attitudinal questions. All attitudinal statements were stated positively. The participants could choose one of the three possible response categories (1 = yes (agree), 2 = no (disagree), 3 = do not know). The practice of the FP was assessed with two statements: do you currently utilize any of the FP methods? Which type of FP methods do you utilize?

Mean scores of knowledge and attitude about FP were calculated to classify the respondents into two groups (knowledgeable and not knowledgeable, good attitude and poor attitude). To calculate the mean score of knowledge, participants who answered *“Yes”* were considered as correctly answered and those who answered *“No”* and *“I do not know”* were considered as not answered correctly. Respondents who scored the mean and above the mean score of the correctly answered questions were classified as knowledgeable, less than the mean score of correct answers were classified as not knowledgeable. To calculate the mean score of attitude, participants who answered “yes/agree” were considered as correctly answered and those who answered “no/disagree” and “I do not know” were considered as not answered correctly. Respondents who scored the mean and above the mean score of attitudinal statements were considered as having a good attitude and less than the mean score as a poor attitude. Concerning family planning practice, it was measured by calculating the percentage of disabled people who currently utilized any of family planning methods. If a married man whose wife was using FP of her choice and he could report that her FP utilization, then he was considered as the current FP user.

### Data processing and analysis

Data was checked for completeness and inconsistencies. Epi-data version 3.1 was used for data entry and data were exported to SPSS version 21. Descriptive statistics were computed. Logistic regression was performed to analyze the data. The bivariate logistic regression analyses were performed between independent and the outcome variables. Those independent variables which were statistically significant in the bivariate model (*p-value < 0.05*) were entered into the multivariable analysis. In the final model, a significant association was declared at a p-value of less than 0.05. And finally, the results were presented in texts and tables with an adjusted odds ratio (AOR) and the corresponding 95% CI.

## Results

### Socio-demographic characteristics

A total of 397 disabled persons were interviewed with a response rate of 96.4%. The mean age of the respondents was 27.7(±7.1SD) years. Most of the respondents (45.2%) had impaired mobility. Fifty-six percent of respondents were female and 48.1% of participants were single. The highest number of study participants were Orthodox Christians (93.0%). A quarter of participants were living alone and 41.1% of disabled persons had no job. Regarding the educational level of the respondents, 48 % (48.5%) of respondents had completed primary education (Table 1).

**Table 1:** Sociodemographic characteristics of respondents in North-shewa zone, Ethiopia, 2019

Socio-demographic characteristics (***N*** = 397)	Categories	N (%)
Form of disability of respondents	Partial mental impairment	13(3.3)
	Hearing impairment	57(14.4)
	Visual impairment	94(23.7)
	Impaired mobility	197(45.2)
	Multiple impairments	53(13.3)
Age	18–30	271(68.1)
	31–40	47(11.9)
	41–50	79(20.0)
Sex	Female	173(43.7)
	Male	224(56.3)
Marital status	Married	142(35.9)
	Single	191(48.1)
	Divorced/widowed	64(15.9)
Religion	Orthodox	369(93.0)
	Muslim	19(4.8)
	Protestant/Catholic	9(2.3)
Educational status	No education	94(23.7)
	Primary education	194(48.9)
	Secondary education	52(13.0)
	College education	57(14.4)
Work status	Had no job	163(41.1)
	Student	63(15.9)
	Employed	57(14.4)
	Lottery seller	41(10.3)
	Others (merchants, daily laborer, beggar, tailor)	73(18.3)
Living condition	With parents	105(26.3)
	With relatives	21(5.2)
	With friends/peers	124(31.4)
	With partner	93(23.5)
	Alone	54(13.6)

### Knowledge and practice of participants about FP methods

As indicated in Table 2, about 85 % of disabled persons had ever heard about FP methods. The most common source of information was the media (television/radio (69.1%). The mean number of FP methods known by respondents was 44.6%. About three-fourth (74.8%) of the respondents knew injectable while withdrawal (18.1%) was the least known traditional FP method. The mean score of side effects reported by participants was 31.5%. Heavy bleeding or irregular bleeding was the most reported side effect of modern contraceptives (35.9%). Overall, 46 % of study participants were knowledgeable about family planning methods. Only a quarter (24.4%) of disabled persons currently utilized any of family planning methods. Of these family planning users, 76(77.6%) of them were females (Table 2).

**Table 2:** The respondents’ knowledge and practice of FP methods in North-shewa zone, Ethiopia, 2019

Variables	Categories	
Have you ever heard about FP methods? (*n* = 337)	Yes	337(84.9)
	No	60(15.1)
What was the source of information for FP? (n = 337)	Television/radio	233(69.1)
	School teachers	134(39.8)
	Health professionals	196(58.2)
	Associations	67(19.8)
	Training	116(34.6)
	Others^a^	99(29.6)
**What is FP (N = 337)**	**Yes**	**No**
Limiting of number of children	174(51.5)	163(48.5)
The spacing of birth intervals	288(85.5)	44(13.2)
Stopping births	85(25.0)	250(74.4)
Do not know	11(3.2)	
**Which type of FP methods did you know? (*n*** **= 337)**	**Yes**	**No**
Oral contraceptive pills	212(62.8)	125(37.2)
Condoms	183(54.4)	154(45.6)
Injectable	252(74.8)	85(25.2)
Implants	191(56.6)	146(43.4)
Intrauterine contraceptive devices	131(38.9)	206(61.1)
Sterilization (male and female)	92(27.4)	245(72.6)
Calendar method	97(28.8)	240(71.2)
Periodic abstinence	132(39.4)	205(60.6)
Withdrawal (coitus interruptus)	61(18.1)	276(81.9)
Lactational amenorrhea method	81(24.1)	256(75.9)
Mean number of methods known	204(60.6)	
**What are the side effects of using family planning?**	**Yes**	**No**
Heavy bleeding or irregular bleeding	121(35.9)	216(64.1)
Absence of menstrual cycle	72(21.3)	265(78.7)
Abdominal cramp	59(17.4)	278(82.6)
Do not know	148(43.8)	189(56.2)
Mean score of side effects known	106(31.52)	
Do you currently utilize any of family planning methods?	98(24.5)	299(75.5)
**Overall FP knowledge level**	**Not knowledgeable**	**Knowledgeable**
	**182(54.0)**	**155(46.0)**

^a^*Parent, friends, magazine, books*

### Attitude of participants towards FP methods

In this study, the calculated mean score of the attitudinal statements was 1.83(±0.39 SD). Forty-four percent (44.4%) of the respondents were above the mean score and they were considered as having a poor attitude towards FP methods (Table 3).

**Table 3:** The respondents’ attitude towards FP methods in North-shewa zone, Ethiopia, 2019

Variables	Agree	Disagree	Do not know
Do you think using FP makes women unhealthy?	143(35.9)	187(47.0)	67(17.0)
Do you think pregnancy must be properly planned?	316(79.6)	37(9.3)	44(11.1)
Do you think pregnancy spaced < 2 years should be avoided by using family planning methods?	214(54.1)	104(26.3)	78(19.6)
Do you think the use of FP methods interfere sexual relationship between husband and wife?	88(22.2)	232(58.5)	77(19.3)
Do you think using modern FP methods causes anger from God?	198(50.0)	150(37.8)	48(12.2)
Do you think FP methods result in infertility to get pregnant later on?	118(29.6)	194(48.9)	85(21.5)
Do you think women are more responsible than men for using modern FP methods?	131(58.1)	114(28.9)	52(13.0)
**Overall family planning attitude level**	**Poor attitude**	**Good attitude**	
	**176(44.4)**	**221(55.6)**	

### Factors associated with knowledge, attitude, and practice of FP methods

In the bivariate model, the sex of the respondents and level of education were statistically significant with the knowledge of FP methods. Besides, age, marital status, and knowledge of FP methods were associated with the utilization of any type of FP methods in the bivariate model. As indicated in Table 4, the results of the multivariate analysis showed that respondents who completed the primary education were three times more likely to have a good knowledge of FP methods than uneducated participants (AOR = 3.31; 95% CI = 1.37, 7.59). Moreover, participants who completed college education were 7 times more likely to have a good knowledge of FP methods than participants who were uneducated (AOR = 7.23; 95% CI = 2.28, 22.06). Moreover, participants who had a good knowledge of FP methods was 1.6 times (AOR = 1.61, CI = 1.27, 16.24) more likely to utilize any type of FP methods than those having poor knowledge of FP methods (Table 4).

**Table 4:** The analyses of factors associated with the knowledge and the current FP methods utilization among respondents in the North-shewa zone, Ethiopia, 2019

**Variables associated with knowledge of FP methods (n = 337)**		**Poor knowledge**	**Good knowledge**	**COR (95% CI)**	**AOR (95% CI)**
Sex	Female	58.4	35.6	1.00	1.00
	Male	41.8	64.4	2.54(1.03,2.77)*	3.12(0.76,8.11)
Educational status	No education	24.6	8.7	1.00	1.00
	Primary education	46.7	54.8	3.21(1.45,7.65)	3.31(1.37,7.59)*
	Secondary education	18.9	11.5	1.71(0.63, 4.83)	1.83(0.65,5.12)
	College education	9.8	25.0	6.96(2.63,19.85)	7.23(2.28,22.06)*
**Variables associated with the current FP utilization (n = 337)**		**Not utilized FP methods**	**Utilized FP methods**		
Age	18–30	68.5	66.7	1.00	1.00
	31–40	10.8	15.2	1.48(1.02,7.65)*	1.71(0.93,3.91)
	41–50	20.7	18.1	0.91(0.51,1.58)	0.80(0.56,2.13)
Marital status	Single	53.2	33.3	1.00	1.00
	Married	30.5	51.5	2.71(1.45,5.00)*	3.41(1.74,6.68)*
	Divorced/widowed	16.3	15.2	1.9(0.64,3.46)	2.11(0.86,5.71)
Respondent’s knowledge on FP	Poor knowledge	77.0	23.0	1.00	1.00
	Good knowledge	68.3	31.7	1.54(1.11,12.50)*	1.61(1.27,16.24)*

1 = Reference, * *p*-value < 0.05

## Discussions

This study highlights interesting insights into the knowledge, attitude, and practice of family planning methods among persons with disabilities in North-shewa zone, Amhara regional state, Ethiopia, unfortunately, there is very limited data and unclear understanding of the knowledge has been problematic. The current study reported the level of knowledge, attitude, and practice of family planning methods among disabled persons who are marginalized groups of the populations. Accordingly, 46 % of study participants were knowledgeable about family planning methods. Likewise, the mean number of family planning methods known by respondents was 44.6%. The injectable was the most known method of modern contraception (74.8%) while withdrawal (18.1%) was the least known traditional FP method. This figure was slightly lower than the study reported in Kampala-Uganda [23]. Even though the knowledge of contraceptive methods among disabled people was not comparable with the general population, findings from the EDHS data also revealed that disabled people’s knowledge on family planning methods was much lower than our result [21]. In Senegal, people with disabilities had very low knowledge about contraception [24]. Women with intellectual disabilities lacked basic knowledge of contraception in Southeast England [25]. This could be due to persons with disabilities commonly have a restricted access to the use of family planning methods, and they are often socially isolated in obtaining FP services.

Although 55 % of our study participants had favorable attitude about modern contraception, only one-fourth (24.5%) of participants currently utilized any method of contraceptives. In line with our finding, the majority of disabled women had a favorable attitude, but the utilization of FP methods by respondents was poor [26–28]. However, our finding was lower than the study conducted in Addis Ababa where 35% of persons with disabilities utilized modern contraceptives [24]. In Nigeria, 34% of physically disabled adolescents utilized modern contraceptive methods [29]. The utilization of FP methods was 39% among people with disabilities in Kampala district, Uganda [23]. This difference is probably because disabled people are discouraged from discussing sexual-related issues in the study area due to some cultural influences.

In the multivariate analysis, participants who had a good knowledge of FP methods were more likely to utilize any type of FP methods than those having poor knowledge. This agreed with a study reported by E Smith et al. [30] where awareness of family planning determined the utilization of modern contraception. Moreover, participants who completed college education were 7 times more likely to have a good knowledge of FP methods than none-educated participants. Similarly, lower education level was associated with lower utilization of family planning among disabilities [27, 28]. This is plausible finding in that education is a foundation for utilization of FP methods among disabled people through developing their knowledge. Moreover, our finding suggested that knowledge of FP have a positive impact on the utilization of FP methods among disabled people. It could be also explained that as the level of education increased, the person’s level of seeking health information also increased which again improves the practice of FP utilization and services.

Despite its strengths, this study has some limitations that must be acknowledged. Although our study had a diverse set of participants, the study focused on disability-support organizations that may miss some people with disabilities in the community. The authors also acknowledge the possibility of information bias created by the sign language interpreters to interpret for respondents who had a hearing impairment, and the limitations associated with self-reported data.

## Conclusions

In this study, the knowledge, attitude, and practice of disabled people about FP methods were relatively low. Due attention should be given to ensure that disabled people are well informed about FP methods through information, education and communication activities.

## Data Availability

All data generated/analyzed during this study are included in this published article. Besides, part of the row datasets will be available from the corresponding author on a reasonable request.

## Abbreviations

AOR: Adjusted Odds Ratio
CI: Confidence Interval
COR: Crude Odds Ratio
FP: Family Planning
SD: Standard Deviation
SPSS: Statistical Package for the Social Sciences
SRH: Sexual and Reproductive Health
